# Extracellular Vesicles and Endocrine Disruption: How Environmental Pollutants Modulate the Loading and Release of Extracellular Vesicles for Cancer Promotion and Progression

**DOI:** 10.3390/ijms27052100

**Published:** 2026-02-24

**Authors:** Sol Buján, Sergio Esquivel-Ruiz, Alicia Olivas-Martínez, Noelia V. Miret, Mariana F. Fernández, Andrea Randi

**Affiliations:** 1 CONICET-Universidad de Buenos Aires, Instituto de Investigaciones Biomédicas (INBIOMED), Laboratorio de Efectos Biológicos de Contaminantes Ambientales, Buenos Aires 1121, Argentina; solbujan00@gmail.com (S.B.); nmiret@fmed.uba.ar (N.V.M.); 2 Departamento de Farmacología, Facultad de Medicina, Universidad de Granada, 18016 Granada, Spain; sesquivel@ugr.es; 3 Instituto de Investigación Biosanitaria (ibs.GRANADA), 18012 Granada, Spain; aolivas@ugr.es (A.O.-M.); marieta@ugr.es (M.F.F.); 4 Centro de Investigación Biomédica (CIBM), Universidad de Granada, 18016 Granada, Spain; 5 Ciber de Epidemiología y Salud Pública (CIBERESP), Instituto de Salud Carlos III, 28029 Madrid, Spain; 6 Departamento de Bioquímica Humana, Facultad de Medicina, Universidad de Buenos Aires, Buenos Aires 1121, Argentina

**Keywords:** extracellular vesicles, endocrine disrupting chemicals, cancer, arsenic, polycyclic aromatic hydrocarbons, bisphenol A, phthalates, particulate matter 2.5, cigarette smoke

## Abstract

Intercellular communication is mediated by extracellular vesicles (EVs), particles released by all cell types that transfer bioactive cargo (proteins, lipids, nucleic acids) to recipient cells, influencing their function. Furthermore, the human population is simultaneously exposed to mixtures of endocrine-disrupting chemicals (EDCs), capable of altering hormonal homeostasis. Epidemiological and experimental evidence, in animal and cellular models, show that EDCs can contribute to the initiation, development, and progression of carcinogenesis. This review analyzes the EDC–EV–Cancer axis, connecting the biology of EVs to environmental toxicology and the processes that lead to tumor development. It has been examined how specific pollutants—arsenic, polycyclic aromatic hydrocarbons, bisphenol A, phthalates, particulate matter 2.5, and cigarette smoke—modify the secretion and content of EVs. These altered EVs may subsequently trigger critical oncogenic mechanisms in recipient cells, including proliferation, angiogenesis, migration, immunosuppression, and metastasis. Specific mechanisms, pathways, miRNAs, and proteins have been identified, following exposure to various EDCs that are capable of modulating cells and the tumor microenvironment to induce carcinogenesis and tumor progression. Therefore, EVs represent a promising platform for investigating the role of exposome in tumor development, serving as a real-time monitoring system that would allow tracking of combined and dynamic human environmental exposure and help in cancer prevention.

## 1. Introduction

Cancer is the leading cause of premature death worldwide, accounting for nearly 10 million deaths in 2020, with the global burden projected to increase by 47% over the next decades—particularly in countries with transitioning economies [1]. The etiology of cancer is increasingly understood through the lens of the “exposome”, a paradigm that encompasses the totality of an individual’s environmental exposures and their biological responses over a lifetime [2]. This framework reframes cancer not merely as a disease of genetic aberrations but as a complex outcome of persistent gene–environment interactions, where environmental carcinogens are recognized as major contributors to the global cancer burden [3]. Central to cancer progression is the dynamic interplay within the tumor microenvironment (TME). This complex ecosystem comprises a diverse cellular compartment—including malignant cells, cancer-associated fibroblasts (CAFs), endothelial cells, and a variety of immune cells (such as tumor-associated macrophages and lymphocytes)—and an extracellular compartment composed of the extracellular matrix (ECM) and a secretome rich in cytokines and growth factors. The communication between these components is paramount for tumor growth, invasion, and metastasis [4]. A primary mode of this intercellular dialogue is mediated by extracellular vesicles (EVs), which function as subcellular messengers, shuttling bioactive cargo between cells to orchestrate local and systemic pathological changes [5]. Concurrently, EVs have been identified as nanoscale vesicles that encapsulate a molecular “fingerprint” of their parent cell, containing a rich cargo of proteins, nucleic acids, and lipids [6]. In the context of cancer, this ability of EVs to mirror the molecular state of their cell of origin endows them with substantial clinical relevance, positioning them as promising minimally invasive diagnostic tools as well as emerging therapeutic platforms [7]. Among the vast array of environmental contaminants, endocrine disrupting chemicals (EDCs) represent a pervasive and insidious class of pollutants with a well-established link to carcinogenesis [8]. This review argues that EDCs may be critical environmental drivers in carcinogenesis, exerting their pro-tumorigenic effects not only through direct cellular toxicity but also by fundamentally altering the biogenesis, cargo, and signaling function of EVs. Evidence indicates that EDCs may use this sophisticated intercellular communication system to induce a pro-inflammatory, pro-proliferative, and pro-metastatic TME, thereby promoting multiple hallmarks of cancer [9]. This new conceptual framework—the ‘EDC–EV–cancer axis’—changes our understanding of environmental carcinogenesis by shifting from a cell-centered model to a systems-level communication network.

It is crucial to note that EVs exhibit a dual role in cancer biology. While this review focuses on their pro-tumorigenic potential under EDC exposure, EVs derived from non-transformed cells or specific immune populations can also exert tumor-suppressive effects and maintain tissue homeostasis. The final pathological outcome depends on the cellular origin and the specific bioactive cargo being transferred [4,6].

EDCs not only damage the clonogenic cell, but probably also force it to secrete ‘toxic messages’ in the form of altered EVs. These messages may spread the damage to both neighbouring and distant cells, amplifying the initial toxic aggression and remodeling the entire tissue microenvironment by altering the stromal cell phenotypes and the biochemical composition of ECM. Consequently, the true carcinogenic potential or tumor progression capacity of an EDC would lie not only in its direct intrinsic toxicity but in its ability to corrupt the organism’s intercellular communication network, a concept that connects the fields of toxicology, cell biology and oncology.

## 2. Extracellular Vesicles: The Messengers of Health and Disease

### 2.1. Biogenesis and Heterogeneity: A Spectrum of Vesicles

EVs are a heterogeneous population of particles wrapped in a lipid bilayer, which do not self-replicate and are released into the extracellular space by virtually all cell types [10]. EVs are broadly classified based on their biogenesis, size, and composition. The main subtypes include exosomes, which are small vesicles (30–150 nm in diameter) of endosomal origin, and ectosomes (also known as microvesicles), which are larger vesicles (50–1000 nm) that bud directly from the plasma membrane [4]. Apoptotic bodies, the largest class, are released during programmed cell death [5]. Due to overlapping physical properties and the technical challenges of isolating pure subpopulations, the International Society for Extracellular Vesicles (ISEV) recommends using the umbrella term “extracellular vesicles” unless a specific biogenic origin can be unequivocally demonstrated [11].

**Figure 1:**
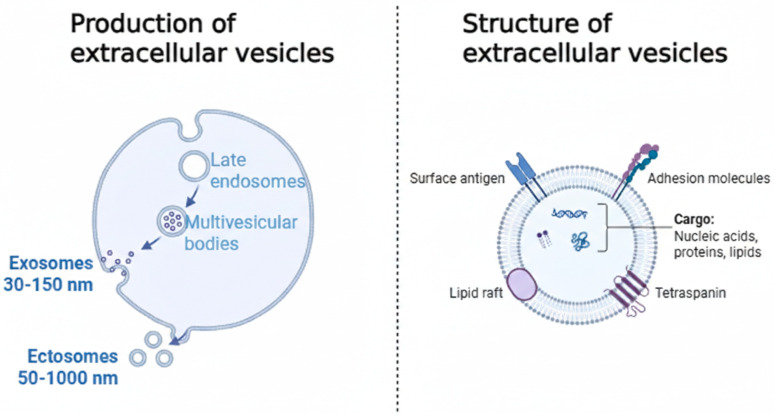
Biogenesis and molecular structure of Extracellular Vesicles (EVs). (**left**) illustrates the two primary pathways of EV production. Exosomes (30–150 nm) originate from the endosomal pathway, where the limiting membrane of Multivesicular Bodies (MVBs) fuses with the plasma membrane to release intraluminal vesicles into the extracellular space. Ectosomes (50–1000 nm), or microvesicles, are formed through the direct outward budding and fission of the plasma membrane. (**right**) depicts the general structure of an EV, consisting of a lipid bilayer membrane enriched with surface proteins such as tetraspanins, adhesion molecules, and antigens. The internal bioactive cargo is selectively packaged and includes nucleic acids (DNA, mRNA, miRNA), proteins, and lipids, which reflect the physiological state of the parent cell.

The biogenesis of exosomes is a complex process initiated within the endosomal pathway. It consists of the internal budding of the late endosomal membrane to form intraluminal vesicles (ILVs) within a multivesicular body (MVB). This process is mediated by both the Endosomal Sorting Complexes Required for Transport (ESCRT) machinery, which recognizes and sorts ubiquitinated cargo, and ESCRT-independent mechanisms involving lipids like ceramide and tetraspanins [4]. The MVB can then either fuse with a lysosome for degradation of its contents or traffic to the plasma membrane, where its fusion results in the release of the ILVs as exosomes [12]. In contrast, ectosomes are formed through the outward budding and fission of the plasma membrane, a process involving cytoskeletal rearrangements and changes in lipid distribution [5]. Figure 1 illustrates the biogenesis and molecular structure of EVs.

### 2.2. The Molecular Cargo: A Representative Part of the Parent Cell

The cargo of EVs is not a random assortment of cellular components but a selectively packaged collection of bioactive molecules that reflects the physiological and pathological state of the parent cell [6]. It is important to highlight that its membranous envelope prevents the degradation of its contents, allowing the EVs to have long-lasting and long-range actions [13]. The cargo includes:

- Proteins: EVs contain a wide variety of proteins, including transmembrane proteins integral to EV structure and function (e.g., tetraspanins such as CD9, CD63 or CD81), cytosolic proteins involved in biogenesis (e.g., Alix, TSG101 or flotillins), and cell-type-specific proteins that can serve as biomarkers [5].
- Nucleic Acids: EVs are potent carriers of genetic content, mainly microRNAs (miRNAs), which can post-transcriptionally regulate gene expression in recipient cells. Messenger RNAs (mRNAs), long non-coding RNAs (lncRNAs), and even fragments of DNA have also been identified within EVs, suggesting multiple avenues for genetic reprogramming [4].
- Lipids: EVs are high in cholesterol, phosphatidylcholine, phosphatidylserine, sphingomyelin, and ceramide. These lipids have been reported to perform various functions in the biogenesis as well as the uptake and mechanisms of action of EVs in receptor cells [14]. Bioactive lipids can also be part of the cargo, acting as signaling molecules themselves [15].

### 2.3. Mechanisms of Intercellular Transfer and Recipient Cell Reprogramming

EVs act as vehicles for intercellular communication by delivering their cargo to recipient cells. This transfer can occur through several mechanisms, including direct fusion of the EV membrane with the plasma membrane of the target cell, or uptake via endocytic pathways such as phagocytosis, macropinocytosis, or receptor-mediated endocytosis [4]. The specificity of this interaction is often guided by receptor–ligand binding between proteins on the EV surface and the recipient cell [5]. Once internalized, the EV cargo is released into the cytoplasm or trafficked through the endo-lysosomal system, where it can modulate the recipient cell’s phenotype by altering gene expression, activating signaling pathways, and reprogramming metabolic processes [16,17].

## 3. Endocrine Disrupting Chemicals as Contributors of Carcinogenesis

EDCs are a heterogeneous group of exogenous chemicals that interfere with the synthesis, secretion, transport, binding, action, or elimination of endogenous hormones in the body [18]. These chemical compounds are non-genetic factors that may be involved in the development or progression of various human diseases, including cancer [19]. Their mechanisms of action are diverse and could include:

- Hormone receptor interaction: Many EDCs act by directly binding to nuclear hormone receptors, such as the estrogen receptor (ER) and androgen receptor (AR) among others, and to membrane hormone receptor, such as G protein-coupled estrogen receptor (GPER) [20]. EDCs can function as agonists, mimicking the effect of the endogenous hormones, or as antagonists, preventing the hormone from binding to its receptor and exerting its effect [21].
- Alteration of hormone levels: EDCs can also disrupt the endocrine system by interfering with the production or metabolism of endogenous hormones [18].
- Epigenetic modifications: A growing body of evidence indicates that EDCs can induce lasting changes in gene expression without altering the DNA sequence itself. These epigenetic modifications, such as DNA methylation and histone modification, could be heritable and are thought to be involved in the developmental origins of adult diseases, including cancer [22].
- Activation of aryl hydrocarbon receptor (AhR): Many EDCs activate or are agonists of AhR, a transcription factor involved in inflammation, proliferation, tumor progression, and chemoresistance [23]. The exposure to AhR agonists stimulates signaling pathways that promote breast cancer development and may contribute to tumor progression [23]. AhR agonists induce different biological mechanisms of action, such as alteration of the cell cycle, proliferation, epigenetic changes, epithelial-to-mesenchymal transition, angiogenic processes, metastasis, chemoresistance, and stem cell renewal [23]. In addition to being involved in the development and progression of breast cancer due to exposure to EDCs, AhR is also involved in other types of cancer, such as prostate [24], colorectal [25], lung [26], skin [27], ovarian [28] and gastric [29]. On the other hand, it is interesting to highlight that in some cell types AhR interacts with other hormone receptors (ERα, GPER and AR) to trigger different carcinogenic characteristics that lead to tumor development [30,31,32,33]. However, it has also been observed that some AhR ligands may exert antiproliferative and antitumor effects [34].

### 3.1. A Focus on Probable Environmental Carcinogens

This review focuses on six classes of EDCs with well-documented environmental prevalence and established or suspected links to cancer:

- Arsenic (As): Exposure to certain heavy metals, at high concentrations or lower concentrations during prolonged periods, may cause a variety of adverse effects in humans and other living organisms [35]. Heavy metals are also persistent compounds due to their bioaccumulation, becoming potentially hazardous pollutants [35]. Arsenic (As), for example, is a metalloid classified in Group 1 (carcinogenic to humans) by the International Agency for Research on Cancer (IARC) [36]. Chronic exposure to As affects millions of people worldwide and is linked to cancers of the liver, lung, skin and bladder [37,38]. Potential sources of As include the consumption and use of contaminated water, the ingestion of contaminated food, smoking, and various occupations (mining) and industrial processes. Some of these sources of exposure appear to be unavoidable, such as the consumption of contaminated groundwater in regions such as Central and South America, India, Bangladesh, and China, where As is found naturally as a geological source [39]. The most common inorganic forms of arsenic are trivalent arsenite (As3+) and pentavalent arsenate (As5+).
- Polycyclic aromatic hydrocarbons (PAHs): These are a large group of persistent organic compounds formed during the incomplete combustion of organic materials (e.g., tobacco, grilled food, petrol, gas, wood, rubbish, etc.). Human exposure to PAHs occurs through complex mixtures of different compounds that, once inside the body, are metabolized through cytochrome P450 family 1, subfamily A, member 1 (CYP1A1),1B1 and EH, CYP-peroxidase and aldo-keto reductase (AKR) pathways, leading to the generation of active carcinogenic compounds (diol-epoxides, radical cations and o-quinones) [40]. Some PAHs, such as benzo(a)pyrene (BaP), are potent carcinogens that exert their effects mainly through AhR activation [15,16]. Chronic exposure to PAHs has also been associated with respiratory diseases, cardiovascular problems and suppression of the immune system [41].
- Bisphenols: Bisphenols are non-persistent organic compounds present mainly in plastic materials. The main route of exposure is through oral ingestion, via contaminated food and materials in contact with food and beverages [42]. The best-known compound in this group is bisphenol A (BPA), a synthetic estrogen whose exposure has been associated with hormone-dependent cancers, such as breast and prostate cancer [12,43]. There is no consensus, however, on whether BPA exposure is carcinogenic to humans; nevertheless, there is extensive experimental evidence from in vitro and in vivo studies, as well as from epidemiological studies, supporting its possible impact on tumor development [44]. The European Union has recently banned the use of BPA in food contact items due to its proven harmful effects on the immune, reproductive and endocrine systems [45]. The widespread use of plastic polymers (polycarbonate) and epoxy resin-based materials (dental) has been linked to an increase in the internal dose of BPA in the population [46].
- Phthalates: Human exposure to these non-persistent organic pollutants is a growing concern worldwide, as they are found in many consumer products (plastics, cosmetics, packaging, etc.). All individuals are exposed to phthalates. Exposure occurs through different routes, including ingestion, inhalation, and dermal contact, with exposure during critical stages of development (e.g., pregnancy) being of particular concern [47]. Some phthalates are suspected of acting as endocrine disruptors, with links to reproductive toxicity and, potentially, urothelial and prostate cancer [48]. Thus, some studies show that human exposure to different phthalates can stimulate pathways leading to carcinogenesis [47,49], with mechanisms of action mediated by the binding of phthalates to AhR [50]. In fact, some phthalates, such as dibutyl phthalate (DBP) and di(2-ethylhexyl) phthalate (DEHP), have AhR agonist activity [51]. European Union regulations (2022) prohibit and regulate phthalates in food packaging [52].
- Particulate matter 2.5 (PM2.5): Particles suspended in polluted air, with an aerodynamic diameter of less than 2.5 μm, originate from combustion sources such as industrial activities, traffic, and the burning of coal and biomass [53]. PM2.5 typically consists of aggregates of smaller carbon particles with mixtures of persistent and non-persistent organic compounds attached to their surface, such as PAHs [54]. Outdoor PM and diesel exhaust particles are classified as Group 1 human carcinogens by the IARC [55]. Several epidemiological studies have observed that living near busy roads (and therefore with higher PM levels) increase the risk of lung cancer [56,57].
- Cigarette smoke: Tobacco use is the leading lifestyle-related risk factor, contributing to the global burden of cancer-related mortality and accounting for approximately 85% of all lung cancer cases [58]. Cigarette smoking is also linked to other types of cancer, such as leukemia, pancreatic cancer, bladder cancer, oral cavity cancer, pharyngeal cancer, laryngeal cancer, esophageal cancer, stomach cancer, liver cancer, kidney cancer, cervical cancer, and ovarian cancer [59]. Cigarette smoke contains more than 4500 different persistent and non-persistent chemical compounds, of which approximately 60 have been classified as carcinogens [60]. Smoking habit also affects the endocrine system [61]. Epidemiological studies show that over 80% of smokers worldwide live currently in low- and middle-income countries [59].

### 3.2. How EDCs Modulate the Secretion and the Content of EVs

The carcinogenic potential of EDCs extends beyond their direct effects on target cells. Many EDCs are potent inducers of chronic inflammation and oxidative stress, two key enabling characteristics of cancer [62]. By perturbing the delicate balance of the TME, EDCs can alter the function of key stromal components; for instance, they can promote angiogenesis by stimulating endothelial cells and modulate the immune response by inducing an immunosuppressive phenotype in macrophages and T cells, creating an environment that is permissive for tumor growth and progression [63].Among the possible actions of the TME induced by exposure to EDCs are the modification of the content and secretion of EVs. Although the mechanisms of action and functions of EVs have been extensively studied, it is unknown whether these functions differ when cells are exposed to different environmental pollutants, such as some EDCs. The central idea of this review is that EDCs exert significant procarcinogenic effects by altering the biogenesis, cargo, and function of EVs. This section summarizes the available scientific evidence linking EDCs, EVs, and cancer for six classes of chemical compounds.

Table 1 summarizes the specific contaminants reviewed, the target cells and signaling pathways involved, the EV loads (RNA, DNA and proteins) and the various induced effects that promote oncogenic processes, oxidative stress, inflammation, proliferation, epithelial–mesenchymal transition (EMT), angiogenesis and metastasis. In some cases, the signaling pathways and/or EV cargo are unknown and are indicated as “Not evaluated”.

#### 3.2.1. Arsenic

##### Modulation of Inflammatory and Proliferative Pathways by Exosomal Onco-miRs

Chronic exposure to inorganic arsenic [As(III)] induces malignant transformation of epithelial cells, causing the secretion of EVs carrying pathogenic miRNA, spreading the carcinogenic phenotype to neighboring normal cells [37,38]. This process shows remarkable tissue-specificity, whereby As(III) leverages distinct signaling pathways to package different onco-miRs tailored to drive relevant carcinogenic hallmarks in different organs.

In the liver, arsenite exposure activates the pro-inflammatory transcription factor nuclear factor kappa B (NF-κB) which leads to the increased expression and selective packaging of miR-155 into EVs. These miR-155-laden EVs are then released and taken up by surrounding normal hepatocytes. The transfer of exosomal miR-155 into recipient cells may induce a potent pro-inflammatory phenotype, characterized by more secretion of cytokines interleukin IL-6 and IL-8 and the subsequent activation of the signal transducer and activator of transcription 3 (STAT3) signaling pathway, creating a microenvironment that promotes the development of hepatocellular carcinoma [37].

Arsenite uses a different signaling cascade in the lung. In human bronchial epithelial (HBE) cells, it stimulates an autocrine/paracrine loop involving IL-6, which activates the STAT3 pathway. This protein drives the transcription of miR-21, another well-known onco-miR, which is packaged into EVs and secreted. When these EVs are internalized by neighboring normal HBE cells, the transferred miR-21 functionally represses the tumor suppressor gene phosphatase and tensin homolog (*PTEN*). Downregulation of *PTEN* unleashes the PI3K/Akt signaling pathway, a master regulator of cell growth, leading to uncontrolled proliferation and promoting lung carcinogenesis [38].

The adaptability of the molecular strategy of As(III) is highly sophisticated, as it alters some of the most relevant signaling pathways in each tissue (NF-κB for inflammation in the liver, STAT3 for proliferation in the lung) to promote the most pertinent cancer hallmarks [9,37,38].

##### Induction of Changes Towards the Epithelial–Mesenchymal Transition via EVs Enriched with Proinflammatory and Oncogenic Factors

Several epidemiological studies consistently show a significant correlation between chronic exposure to As and an increased risk of lung, bladder, and skin cancer [64]. Exposure to As has also been associated with prostate cancer [64]. Thus, it has been previously reported that arsenic-transformed prostate epithelial (CAsE-PE) cells can recruit prostate stem cells to acquire a cancer stem cell (CSC) phenotype through the secretion of soluble factors [65]. In this regard, Ngalame et al. [65] postulate that CAsE-PE cells recruit stem cells for a CSC-like phenotype by the production of EVs, since CAsE-PE secretes 700% more EVs than non-transformed parental cells. EVs from CAsE-PE cells are enriched with various molecules, including oncogenic factors [(Kirsten rat sarcoma (KRAS), neuroblastoma Ras (NRAS), vascular endothelial growth factor (VEGF), MYB proto-oncogene, and epidermal growth factor receptor (EGFR)], inflammation-related factors [cyclooxygenase-2 (COX-2), IL-1β, IL-6, transforming growth factor-β (TGF-β), and tumor necrosis factor-α (TNF-α)], and apoptosis-related factors [caspase-7 (CASP7), caspase-9 (CASP9), and B cell lymphoma 2 (BCL2)]. Stem cells cultured in the presence of EVs derived from CAsE-PE exhibited increased metalloproteinase (MMP) activity and showed morphological changes towards EMT, suggesting EV-mediated transformation. These findings suggest that As alters both the quantity and cargo of EVs, deepening our knowledge of the mechanism by which As modifies stem cell dynamics and modulates the TME during carcinogenesis by inducing the secretion of pro-inflammatory cytokines (IL-1β, IL-6, TNF-α) and increasing the activity of MMPs, which degrade the ECM to facilitate cell migration [65].

#### 3.2.2. Polycyclic Aromatic Hydrocarbons (PAHs)

##### Triggering Oxidative Stress Cascades Through Pro-Oxidant EVs

PAHs induce a significant increase in EV release in hepatocytes, although the mechanism involved depends on each chemical compound and its affinity for the AhR [15]. Thus, PAHs with high-affinity, such as BaP and dibenzoanthracene (DBA), activate the AhR signaling pathway leading to the induction of CYP1 enzymes, which, by metabolizing PAHs, would also cause cellular cholesterol depletion [15]. The reduction in cholesterol increases the fluidity of the plasma membrane, facilitating budding and the release of EVs [15]. In contrast, PAHs with low AhR affinity, such as pyrene, stimulate EV release through an alternative pathway involving the constitutive androstane receptor [15].

Apart from these divergent upstream triggers for EV release, the response of cells converges on the packaging of a common, highly toxic cargo. Hepatocytes exposed to PAHs load their EVs with a specific pro-oxidant payload: activated subunits of nicotinamide adenine dinucleotide phosphate (NADPH) oxidase and the iron-storage protein ferritin, which is enriched with iron [16]. These EVs act as veritable oxidative stress bombs. After being taken up by healthy receptor hepatocytes, they are transported to the acidic environment of the lysosome, within which the iron is released. This free iron then catalyzes the Fenton reaction, using hydrogen peroxide generated by the co-transported NADPH oxidase to produce highly reactive and damaging hydroxyl radicals, inducing lipid peroxidation of the lysosomal membrane, leading to its permeabilization. This lysosomal breach triggers a downstream cascade of mitochondrial damage and ultimately induces apoptosis in the recipient cell [16]. The cell then attempts to expel the harmful pro-oxidant components, but this “waste disposal” becomes a weapon that spreads oxidative damage throughout the tissue, creating a chronic stress field conducive to carcinogenesis.

##### Remodeling of the Tissue Microenvironment to Favor Tumor Colonization and Metastasis

The carcinogenic damage caused by PAHs goes beyond inducing local liver toxicity. Some PAHs, such as BaP, appear to exploit EVs to modify distant tissues and establish pre-metastatic niches that would favor tumor colonization long before the tumor cells themselves arrive [66]. For example, EVs can travel from the hepatocellular carcinoma (HCC) to the lung, where they are selectively uptaken by fibroblasts [66]. Within fibroblasts, the circular RNA (circRNA) circ_0011496 derived from EVs functions as a competitive endogenous RNA (ceRNA) with the ability to inhibit miR-486-5p [66]. In addition, circRNA is an abundant and stable class with regulatory functions in EVs. This suppression triggers the expression of Twinfilin-1 (TWF1), upregulating MMP9 synthesis. This molecular cascade provokes the differentiation of normal lung fibroblasts into activated Cancer-Associated Fibroblasts (CAFs) [66]. These BaP-educated CAFs can remodel the lung microenvironment by upregulating MMP9, which alters the ECM structure, and by inducing inflammation and angiogenesis to build a permissive “pre-metastatic niche” that actively recruits circulating tumor cells, thereby facilitating specific metastasis (organotropic metastasis) of liver cancer to the lung.

#### 3.2.3. Bisphenol A (BPA):

##### BPA Drives Oral Squamous Cell Carcinoma (OSCC) Progression

BPA has been linked to the progression of OSCC. Thus, exposure to BPA appears to increase the proliferation, migration, and invasion of OSCC cells, while promoting stemness through the gamma-aminobutyric acid type B receptor subunit 1 (GABBR1)/MEK/ERK signaling pathway and EV-driven immunomodulation. Specifically, He et al. [67] identified GABBR1 as a key gene in OSCC and analyzed its expression patterns and clinical significance. These same authors also characterised the binding interaction between BPA and GABBR1. They found that BPA exposure also induced M2 macrophage polarisation through EV communication, contributing to an immunosuppressive TME [67].

##### Dysregulating EV Secretion in Breast Cancer

BPA alters both the biogenesis and secretion mechanisms of EVs. Its impact extends beyond modifying vesicular cargo by increasing EV quantity, establishing a positive feedback loop that saturates the local microenvironment with pro-tumorigenic signals [12]. In MCF-7 breast cancer cells (ER+/PR+), BPA exposure may induce the downregulation of the tumor suppressor microRNA miR-26b [12]. This reduction leads to the upregulation of the protein Rab31, which functions as a molecular switch. Rab31 inhibits the fusion of multivesicular bodies (MVBs) with lysosomes—preventing the degradation of their contents—and redirects them toward the plasma membrane for fusion and release [68]. This increased rate of EV secretion is directly responsible for the enhanced proliferation and migration of MCF-7 cells. The causal relationship is confirmed by the use of the pharmacological agent GW4869, which reverses the BPA-induced malignant phenotype by inhibiting EV release [12].

#### 3.2.4. Phthalates

##### Modulating the PTEN/PI3K/Akt Tumor Suppressor Pathway

A recent biomonitoring study conducted in healthy, non-smoking Italian men found a significant positive correlation between urinary concentrations of certain phthalate metabolites, such as mono-isobutyl phthalate (MiBP) and mono-n-butyl phthalate (MBP), and the expression levels of specific exosomal miRNAs (miR-202 and miR-543) in EVs isolated from the same urine samples [48]. These urinary EVs, secreted by the kidney, urothelium, and prostate cells, serve as effective ‘liquid biopsies’ of tissues directly exposed to excreted phthalate metabolites. Notably, these vesicles reflect early molecular reprogramming and could serve as biomarkers of early biological response. Although no neoplasms were observed in this study, the downregulation of the tumor suppressor gene *PTEN* by miR-202 and miR-543 could initiate oncogenic signaling long before clinical symptoms appear [48].

Since *PTEN* is a master regulator that acts as a critical brake on the PI3K/Akt signaling pathway—controlling cell growth, proliferation, survival, and metabolism—the delivery of these miRNAs to recipient cells suggests a plausible mechanism by which phthalates contribute to pathway dysregulation and a pro-tumorigenic state [48]. Consequently, this association highlights the potential of urinary EVs as a non-invasive monitoring system for environmental exposure and subsequent carcinogenesis in the male urogenital tract. These findings underscore the utility of urine samples in basic science and toxicology to detect the impact of pollutants on metabolic pathways, though further studies in other human populations are needed to verify these effects [48].

#### 3.2.5. Particulate Matter 2.5 (PM2.5)

##### EVs of Human Bronchial Epithelial Cells Treated with PM2.5 Induce Migration and Invasion in Lung Tumor Cells and Pulmonary Metastases in Mice

Yu et al. [69] cultured human bronchial epithelial (HBE) cells exposed or not exposed to PM2.5 (25 µg/mL), extracting EVs from the two experimental groups. The EVs were used to treat mice and/or lung tumor cells. Specifically, they first injected human lung tumor cells (A-549) into the caudal vein of nude mice with the aim of inducing metastasis. After 4 days, the animals received EVs from HBE cells via the caudal vein twice a week for 30 days. The results showed that mice treated with EVs derived from HBE cells exposed to PM2.5 (25 µg/mL) exhibited larger metastatic nodules and a significantly higher relative metastatic region compared to the other groups [69]. To evaluate the mechanism underlying the increased metastatic potential of mice treated with EVs from exposed cells, in vitro assays were performed. They used lung tumor cell lines (A-549 and H-1975) treated with EVs, observing greater migration and invasion in cells treated with EVs derived from HBE cells exposed to PM2.5 compared to cells treated with EVs from unexposed cells.

In addition, Yu et al. [69] studied the proteins involved in the EMT of tumor cells (A-549 and H-1975). They observed that tumor cells treated with EVs derived from HBE cells exposed to PM2.5 (25 μg/mL for 20 passages) altered EMT marker proteins, reducing E-cadherin and increasing N-cadherin and zinc finger E-box-binding homeobox 1 (ZEB1). Cells treated with EVs from HBE cells not exposed to PM2.5 showed no differences in these markers. These findings suggest that EVs from HBE cells exposed to PM2.5 contribute to increasing the malignancy of lung tumor cells by promoting migration, invasion, and EMT. The increase in migration and invasion appears to depend on the c-Jun N-terminal kinase (JNK) pathway [69].

Current knowledge also allows us to understand the effects of chronic exposure to low doses, a scenario that more accurately mimics the environmental conditions of the general population. Complementing these findings, Yu et al. [70] investigated the effect of exposing normal bronchial epithelial cells (BEAS-2B) to low concentrations of PM2.5 (5 μg/mL) over a prolonged period (30 passages). Carcinogenicity was found to be mediated by a specific alteration in the microRNA load of secreted EVs. Specifically, miR-196b-5p was identified as the most significantly upregulated miRNA in EVs derived from cells chronically exposed to PM2.5 [70]. It is important to note that EVs are internalized by both tumor cells and adjacent healthy epithelial cells. Once inside the recipient cell, miR-196b-5p binds directly to the mRNA of *CDH1* (the gene encoding E-cadherin), causing its degradation and a subsequent decrease in this protein. This finding is significant because it establishes a dual mechanism: PM2.5-induced EVs not only promote malignancy in existing cancer cells but also induce an invasive phenotype and EMT in adjacent normal bronchial epithelial cells, spreading damage throughout healthy lung tissue [70]. In vivo trials also showed that the inhibition of miR-196b-5p through modified EVs was able to reverse invasive capacity and suppress lung metastasis [70].

##### Chronic PM2.5 Exposure Induces Atypical Hyperplasia and Alters EV miRNA Profiles Associated with Tumorigenesis

To deepen the transcriptomic understanding of PM2.5-induced exosomal alterations, Wang et al. [71] designed two chronic exposure protocols: an in vitro model with human A549 lung carcinoma cells (exposed to 100 µg/mL of PM2.5 for 5 passages) and an in vivo model with mice administered an intratracheal suspension of 20 mg/kg of PM2.5 every 3 days for more than 90 days. The in vivo findings revealed that chronic instillation of PM2.5 induced pathological changes, such as atypical hyperplasia of the bronchial epithelium and massive macrophage infiltration [71]. The authors demonstrated significant downregulation of E-cadherin and upregulation of vimentin in the lung tissues of exposed mice as well as in A549 cells, consistent with the EMT phenotypes observed in other studies [69,70].

Stimulation with PM2.5 appears to induce the release of EVs with a clearly altered miRNA profile. Thus, through RNA sequencing, Wang et al. [71] identified 36 differentially expressed miRNAs (30 upregulated and 6 downregulated) in EVs from exposed cells compared to controls. They observed significant downregulation of miR-29b-2-5p, miR-193b-5p, and miR-320c, along with upregulation of miR-100-5p and miR-125b-5p [71]. Bioinformatic analysis suggested that these dysregulated exosomal miRNAs were closely related to tumor development; for example, downregulation of miR-29b-2-5p can reduce p53 expression, affecting cell cycle and apoptosis [72], while alteration of miR-193b-5p modulates migration and invasion in cancers [73].

To sum up, different studies indicate that exposure to PM2.5, at low and high doses, reprograms the bronchial epithelium to secrete EVs with a diverse repertoire of oncogenic miRNAs (miR-196b-5p, miR-100-5p) and depletes tumor suppressors (miR-29b-2-5p), inducing potent EMT and pro-metastatic phenotype.

#### 3.2.6. Cigarette Smoke

##### Altered EV-miRNAs Expression in Early Stages of Lung Cancer

Carcinogens in tobacco smoke appear to modulate cell-to-cell communication, which significantly affects EV-mediated signaling. In addition to stimulating EV release, tobacco smoke can also alter the molecular cargo carried by EVs, including non-coding RNAs (ncRNAs) [74]. There is growing evidence to suggest that tobacco can induce lung carcinogenesis by altering the miRNA levels of EVs [75,76]. Thus, in vitro studies show that human bronchial epithelial (HBE) cells exposed to cigarette smoke increase the production of exosomal miR-21. This oncomiR promotes angiogenesis by increasing VEGF levels in normal HBE recipient cells, mediated by the STAT3 signaling pathway [75]. The impact of tobacco on miR-21 levels in EVs has also been corroborated in in vivo models, with smokers having significantly higher serum miRNA levels than non-smokers [75,76]. In murine models, it has also been observed that 4-(methylnitrosamino)1(3-pyridyl)-1-butanone (NNK), a chemical derived from nicotine, alters the expression of miR-206 and miR-133b, and that these altered EV-miRNAs are upregulated in the early stages of NNK-induced lung carcinogenesis [77]. Therefore, the available scientific evidence suggests that tobacco-induced EV-miRNA signaling may contribute significantly to lung carcinogenesis or potentially serve as a biomarker for it.

In the same context, an in vitro study observed that human airway epithelial cells exposed to tobacco smoke release EVs with altered miRNA and piRNA [RNA interacting with PIWI proteins (P-element-induced wimpy testis)] loads compared to unexposed cells. Specifically, an upregulation of miR-3913-5p, miR-574-5p, and miR-500a-5p was observed, while miR-618 levels were reduced [78]. Deregulation of other miRNAs—let-7e, let-7g, and miR-26b—was also observed in EVs isolated from bronchoalveolar lavage fluid of smokers compared to non-smokers [79]. It is therefore plausible that changes induced by exposure to tobacco smoke in the ncRNAs of EVs may influence multiple biological processes related to the carcinogenesis of recipient cells.

**Table 1:** Summary of the mechanisms by which EDCs modulate EV biogenesis and cargo to drive cancer progression.

EDC Class	Biological Source	Key Signaling Pathway Altered	Key EV Cargo/Secretion Change	Downstream Effect on Recipient Cell	Reference
Arsenic	Human liver epithelial cells	NF-κB	↑ miR-155	Pro-inflammatory phenotype (↑ IL-6, IL-8)	[37]
	Human bronchial epithelial cells	IL-6/STAT3	↑ miR-21	Proliferation (via PTEN suppression)	[38]
	Prostate epithelial cells	“Not evaluated”	KRAS, NRAS, VEGF, EGFR, COX-2, IL-6, TGF-β, TNF-α, BCL-2	↑ MMP activity ↑ Epithelial–mesenchymal transition	[64]
PAHs	Rat hepatocytes	AhR (BaP, DBA)/CAR (PYR)	↑ Ferritin (iron) & NADPH oxidase	Oxidative stress Lipid peroxidation	[15,16]
	Hepatocellular carcinoma cells	circ_0011496 EVs inhibit miR-486-5p that induces ↑ TWF1 + MMP9 in lung	↑ circ_0011496	Lung fibroblasts → CAF ↑ Inflammation ↑ Angiogenesis ↑ Pre-metastatic niche in lung	[66]
BPA	Oral squamous carcinoma cells	GABBR1/MEK/ERK	“Not evaluated”	Macrophage polarization, ↑ Proliferation, migration, invasion, tumor progression	[67]
	Human breast cancer cells (MCF-7)	miR-26b/Rab31	↑ exosome secretion rate	↑ Proliferation, migration	[13,68]
Phthalates	Human urine samples	Associated with target pathways	↑ miR-202 & miR-543 in urine	PTEN/PI3K/Akt pathway dysregulation	[48]
PM2.5	Human bronchial epithelial (HBE) cells	(a) Wnt/β-catenin signaling pathway (b) JNK pathway	“Not evaluated”	(a) EVs from HBE cells treated with PM2.5 inoculated in mice, ↑ Metastasis in lung (b) EVs from HBE cells treated with PM2.5 in tumor lung cells, ↑ Migration, Invasion	[69,71]
	Normal bronchial epithelial cells	“Not evaluated”	↑ miR-196b-5p	- Tumor cells: ↑ Malignancy - Adjacent normal bronchialepithelial cells: ↑ Invasion and EMT	[70]
	A549 Lung adenocarcinoma cells	“Not evaluated”	↑ miR-100-5p, miR-125b-5p; ↓ miR-29b-2-5p, miR-193b-5p, miR-320c	EMT induction (↓ E-cadherin, ↑ Vimentin); ↑ migration, invasion	[71]
	In vivo model in mice		“Not evaluated”	Atypical hyperplasia of bronchial epithelium, macrophage infiltration. EMT induction (↓ E-cadherin, ↑ Vimentin)	
Cigarette smoke	Human bronchial epithelial cells	STAT3 signaling	↑ miR-21	Angiogenesis (↑ VEGF)	[75]
	(a) Human airway epithelial cells (b) Bronchoalveolar lavage fluid of smokers	“Not evaluated”	(a) ↑ miR-3913-5p, miR-574-5p, miR-500a-5p (b) ↓ miR-618, let-7e, let-7g, miR-26b	Pro-carcinogenic cellular reprogramming and induction of preneoplastic status	[78,79]

Abbreviations: AhR: Aryl hydrocarbon Receptor; Akt: Protein Kinase B; BPA: Bisphenol A; CAF: Cancer-Associated Fibroblast; CAR: Constitutive Androstane Receptor; CDH1: Cadherin-1 (E-cadherin); circ_0011496: Circular RNA 0011496; COX-2: Cyclooxygenase-2; EDCs: Endocrine Disrupting Chemicals; EGFR: Epidermal Growth Factor Receptor; EMT: Epithelial–Mesenchymal Transition; ERK: Extracellular signal-Regulated Kinase; EVs: Extracellular Vesicles; GABBR1: Gamma-Aminobutyric Acid Type B Receptor Subunit 1; HBE: Human Bronchial Epithelial cells; IL-6/8: Interleukin-6/8; JNK: c-Jun N-terminal Kinase; MEK: Mitogen-activated protein kinase kinase; miRNA: Micro-RNA; MMPs: Matrix Metalloproteinases; NADPH: Nicotinamide Adenine Dinucleotide Phosphate; NF-κB: Nuclear Factor kappa B; PAHs: Polycyclic Aromatic Hydrocarbons; PI3K: Phosphoinositide 3-kinase; PM2.5: Fine Particulate Matter with a diameter ≤ 2.5 µm; PTEN: Phosphatase and Tensin homolog; STAT3: Signal Transducer and Activator of Transcription 3; TGF-β: Transforming Growth Factor beta; TNF-α: Tumor Necrosis Factor alpha; TWF1: Twinfilin-1; VEGF: Vascular Endothelial Growth Factor. ↑ enhance; ↓ reduce; → it transforms into.

### 3.3. Functional Consequences: How EDC-Modified EVs Generate Preneoplastic Characteristics

Specific molecular alterations induced by EDCs on EV signaling pathways result in the promotion of several well-established hallmarks of cancer [9]. This section explains some of these mechanisms.

#### 3.3.1. Sustaining Proliferative Signaling and Evading Growth Suppressors

Two of the fundamental hallmarks of cancer cells are their ability to sustain chronic proliferation and evade normal growth-suppressive signals [1]. Tumor-derived EVs are known to enhance this cancer cell viability, either by diminishing pro-apoptotic signals within donor cells or by reinforcing anti-apoptotic pathways in recipient cells [80]. The mechanisms employed by As and phthalates, for example, are a clear example of how EDC-modified EVs contribute to these processes. Both As and some phthalates induce the secretion of EVs carrying miRNAs (such as miR-21, miR-202, miR-543) that converge and suppress PTEN [38,48]. By neutralizing this critical cellular brake, EVs release the PI3K/Akt pathway, a potent pro-proliferative and pro-survival signal in recipient cells [38]. Furthermore, exposure to BPA significantly increases the volume of EV secretion in breast cancer cells, amplifying autocrine and paracrine growth factor signaling and facilitating uncontrolled proliferation [12]. It would therefore appear that different classes of EDCs act through a common and critical mechanism of environmental carcinogenesis mediated by EV-miRNAs.

#### 3.3.2. Inducing Chronic Inflammation and Oxidative Stress

The TME is often characterized by chronic inflammation and oxidative stress, known as powerful drivers of tumorigenesis [81]. Findings on arsenic and PAHs show that EDC-modified EVs would mediate these processes. For example, arsenite-induced EV miR-155 acts as a mobile inflammatory signal, instructing neighboring hepatocytes and resident macrophages (Kupffer cells) to produce pro-inflammatory cytokines (such as TNF-α and IL-6), creating a self-sustaining inflammatory loop [37]. Similarly, PAH-induced EVs function as vectors of oxidative stress, delivering the necessary molecular machinery (iron and NADPH oxidase) to generate reactive oxygen species (ROS) in recipient cells [16]. This oxidative damage extends beyond the initially exposed cells, creating a “field effect” of chronic tissue injury and inflammation. In this process are involved the recruitment of neutrophils and the oxidative modification of the ECM proteins, creating a stromal state highly conducive to malignant transformation [63].

#### 3.3.3. Promotion of Angiogenesis and an Immunosuppressive TME

Angiogenesis is defined as the process involving the formation of new capillaries that sprout from the pre-existing vasculature [82]. Chronic and sustained angiogenesis is another recognized hallmark of cancer, which plays a vital role in tumorigenesis by promoting continuous tumor proliferation, ensuring the supply of oxygen and nutrients, and facilitating the removal of metabolic waste [83]. The release of EVs by tumor cells significantly contributes to this process [84]. Scientific evidence shows that these EVs can substantially enhance neovascularization by activating vascular endothelial cells, underscoring the critical interplay between cellular communication and vascular development within the TME [85]. For example, exposure of HBE cells to tobacco smoke condensate increases the generation and secretion of miRNA-21 in EVs. This miRNA stimulates angiogenesis by inducing endothelial cell proliferation and migration through enhanced VEGF expression within recipient cells, a process dependent on the STAT3 pathway [75]. Mu et al. [66] found that CAFs educated by exposure to circ_0011496-laden EVs from liver BaP-exposed tumor cells could remodel the lung microenvironment. Specifically, these CAFs alter the deposition of ECM components like collagen and fibronectin, inducing inflammation and angiogenesis and creating a permissive “premetastatic niche”.

Malignant cells, in contrast to their normal counterparts, are characterised by generating higher amounts of EVs. Tumor-derived EVs have a substantial capacity to influence both the local and distal TME [4,86]. Furthermore, these EVs can encapsulate different bioactive components that engage with specific immune cell populations within the TME, thereby regulating cancer-related phenomena like immunosuppression and metastasis [80]. For instance, the EV cargo may include molecules such as Programmed Death-Ligand 1 (PD-L1) and TGF-β. These mediators act directly on cytotoxic CD8+ T cells to inhibit their activity and promote the differentiation of Regulatory T cells (Tregs), which are primary drivers of immune evasion [87]. Exposure to BPA, for example, triggers the polarization of monocytes into M2 macrophages via EV signaling, contributing to the establishment of an immunosuppressive TME characterized by a pro-tumorigenic cytokine profile [67].

#### 3.3.4. Activating Migration, Invasion, and Metastasis

Metastasis involves local invasion, intravasation, survival in circulation, and colonization of distant organs, and is the leading cause of cancer-related mortality [86]. EVs are now recognized as a key part of this process, and evidence suggests that EDCs may enhance their pro-metastatic functions.

EMT, a crucial early step in metastasis, is a process induced by EVs in which tumor epithelial cells lose their polarity and cell–cell adhesion and acquire migratory and invasive properties [88]. For example, EVs derived from highly metastatic lung cancer cells are enriched with the mesenchymal protein vimentin. When these vimentin-laden EVs are taken up by non-malignant human bronchial epithelial cells, they induce vimentin expression and trigger an EMT phenotype, conferring greater migratory and invasive capabilities on the recipient cells [89]. Several of the studies discussed in this review indicate that exposure to different environmental pollutants stimulates the release of EVs that induce EMT characteristics; for example, the release of EVs enriched in oncogenic and inflammatory factors (VEGF, EGFR, COX-2, IL-6, TGF-β, TNF-α) following exposure of prostate epithelial cells to As [66]. Stem cells treated with those EVs underwent changes toward EMT [65]. Similarly, exposure of OSCC cells to BPA induces enhanced migration and invasion, stimulating the malignant characteristics of these tumor cells [68]. Furthermore, exposure of HBE cells to PM2.5 promotes the secretion of EVs that contribute to increased cell migration, invasion and EMT of lung cancer cells [70].

Beyond local invasion, EVs play a systemic role in preparing distant organs for the arrival of circulating tumor cells, a process known as pre-metastatic niche formation [90]. Tumor-derived EVs travel through the bloodstream and induce ECM remodeling, recruiting pro-tumorigenic immune cells and creating an overall immunosuppressive environment at future metastatic sites [91]. Several examples have already been discussed of how the EDC-EV pathway can stimulate the transformation of tissues where tumor cells settle to generate metastatic nodules. For example, after exposure to BaP, lung fibroblasts are transformed into CAF through the uptake of EVs with circ_0011496 from hepatocellular carcinoma, building permissive pre-metastatic niches in the lung [65]. Yu et al. [69] also reported a higher number of pulmonary metastatic nodules in mice treated with EVs derived from HBE cells exposed to PM2.5. The available evidence therefore indicates that exposure to EDCs would alter the load and volume of circulating EVs, and that primary tumors exposed to EDCs could release EVs with a greater capacity to induce EMT and form premetastatic niches, increasing the likelihood of successful metastatic colonization in distant organs.

### 3.4. Clinical and Toxicological Implications

The discovery that EVs are released into all major body fluids and carry molecular cargo that reflects their cellular origin has positioned them as a promising source for non-invasive “liquid biopsies” [6]. The relationship between urinary phthalate levels and specific exosomal miRNA profiles provides a proof for this application in environmental health [48]. Such EV-based biomarkers could serve as early biological effect markers. Thus, the identification of individuals with altered EV-miRNA profiles could enable risk stratification and intervention prior to the clinical manifestation of the disease. The rich and heterogeneous nature of EV cargo comprising proteins, lipids, and multiple classes of nucleic acids also allows for the development of multi-component biomarker panels [4].

Investigating EVs in response to diverse environmental factors may yield novel mechanistic insights into their involvement in tumorigenesis. EVs not only serve as a repository for tumor cellular information, but can also mediate intercellular communication, driving cancer progression. Furthermore, comprehensive characterization of the cargo and functionalities of tumor-derived EVs would enable us to circumvent current clinical limitations and enhance oncological patient prognosis. Defining EVs as targets for therapeutic strategies or utilizing tumor EVs as biomarkers could significantly accelerate the development of early screening methodologies across various cancer types.

**Figure 2:**
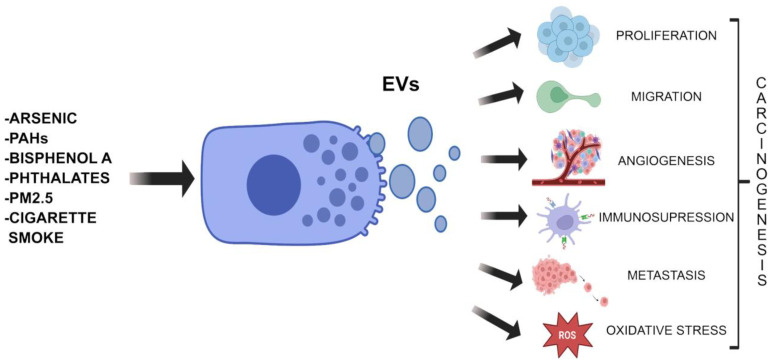
Schematic of the EDC–EV–Cancer axis. Exposure to different environmental pollutants, such as arsenic, PAHs, bisphenols, phthalates, PM2.5, or tobacco smoke, with endocrine disruptive activity, stimulates various signaling pathways that modulate the expression of different genes, miRNAs and lncRNAs, and the production of lipids, and proteins. All these molecules will become part of new EVs that will be secreted and impact target cells, promoting procarcinogenic processes (proliferation, oxidative stress, migration, invasion, angiogenesis, immunosuppression, and metastasis).

The studies analyzed in this work suggest that EVs may act as signal carriers between cells stressed by exposure to EDCs, which promote the progression of carcinogenesis. Exposure to carcinogens activates several mechanism of actions and lead to differential modulation of the expression of certain genes and proteins, releasing EVs that transport integrins, miRNAs, lncRNAs, cytokines, or chemokines that transform the cellular microenvironment. Overall, the studies highlight the emerging role of EVs in inducing carcinogenic changes due to the daily exposome. Figure 2 illustrates a schematic of the impact of EDCs on tumor or non-tumor cells, stimulating the secretion of EVs that influence other target cells, promoting various mechanisms that lead to increased malignancy and/or carcinogenesis. Therefore, circulating EVs in the bloodstream represent an excellent platform for investigating the exposome across various disease contexts, particularly carcinogenesis. Since the sources and magnitude of environmental exposure fluctuate over time, EVs could serve as a real-time monitoring system of these dynamic changes. Furthermore, EVs can potentially encapsulate a molecular signature of accumulated pollutants, making them trackable in both retrospective archival biological samples and prospective clinical cohorts.

## 4. Summary

The convergence of evidence from studies analyzing the consequences of exposure to arsenic, PAHs, BPA, phthalates, PM2.5, or cigarette smoke provides a solid foundation for a new paradigm in environmental carcinogenesis. The EDC-EV interface probably represents a fundamental and powerful mechanism by which environmental exposures may drive cancer. This review establishes that some EDCs are not mere cellular toxins that cause localized damage but sophisticated saboteurs that disrupt the intricate network of intercellular communication in organisms. They could reprogram cells to secrete EVs with pro-proliferative, pro-inflammatory, and pro-metastatic signals, effectively turning a natural communication system into a conduit for disease propagation. Recognition of this EDC–EV–Cancer axis opens up transformative avenues for clinical oncology and public health. It provides a new framework for risk assessment, shifting the focus from simple exposure measurement to the identification of early effect biomarkers. Furthermore, it allows the incorporation of promising, highly specific liquid biopsies based on EV cargo for early diagnosis and disease monitoring. Finally, it reveals a landscape of new therapeutic targets aimed not at directly killing cancer cells but rather at cutting off the corrupt communication lines that fuel their growth and spread.

In our view, the content of this review may be useful in both toxicology and oncology. Studying how exposure to different EDCs impacts EVs will allow us to: a) expand our knowledge of the mechanisms of action of environmental pollutants; b) implement primary and secondary prevention strategies (liquid biopsies) in cancer; and even c) evaluate how pollutants can modulate antitumor therapies. It is recommended that further in vitro and in vivo studies be conducted to assess the protein, miRNA, and lipid content of secreted EVs, in addition to monitoring exposure to EDCs in biomonitoring studies. This information will be a powerful tool for understanding the mechanisms of action of EDCs and for disease prevention.

## 5. Future Directions

### 5.1. Validation in Large-Scale Human Cohorts:

Most current data linking EDCs to altered EV cargo comes from in vitro or animal models, with limited human biomonitoring studies. Future research should prioritize longitudinal epidemiological studies to validate specific EV-associated biomarkers in diverse populations. Establishing a direct correlation between urinary or blood levels of EDC metabolites and specific EV signatures could solidify the use of EVs as non-invasive liquid biopsies for early risk stratification.

### 5.2. EVs as Dynamic Exposome Trackers

The concept of “exposome” implies exposure to mixtures of environmental pollutants that fluctuate over time. Since EVs reflect the real-time physiological state of the parent cell, future technologies should aim to develop multi-component biomarker panels. This would allow EVs to serve as a monitoring system to track the actual, combined, and dynamic human exposome and identify the molecular signatures (proteins, lipids, and nucleic acids) of EDCs before the clinical onset of cancer.

### 5.3. Deciphering Cargo Sorting Mechanisms

While we know EDCs alter EV cargo, the precise molecular machinery by which these chemicals modify the sorting process remains incompletely understood. It is crucial to investigate how EDCs influence the ESCRT-dependent and independent pathways to selectively package pathogenic miRNAs, lncRNAs, lipids or proteins. Elucidating these specific sorting mechanisms could reveal novel targets to, for example, prevent the packaging of “toxic messages”.

### 5.4. Translating Therapeutic Strategies to the Clinic

The therapeutic potential of disrupting the EDC-EV communication network is promising but requires rigorous clinical evaluation. Future drug development should focus on agents that can safely inhibit EV secretion, such as inhibitors of neutral sphingomyelinase 2 (e.g., GW4869) or Rab GTPases, without compromising physiological intercellular communication. Additionally, strategies to neutralize pathogenic cargo must be optimized for systemic delivery and specificity to the TME.

## Data Availability

No new data were created or analyzed in this study. Data sharing is not applicable to this article.

## Abbreviations

AhR	Aryl Hydrocarbon Receptor
AKR	Aldo-keto reductase
AR	Androgen Receptor
BaP	Benzo(a)pyrene
BPA	Bisphenol A
CAF	Cancer-Associated Fibroblasts
CAR	Constitutive Androstane Receptor
CDH1	Cadherin-1
CYP1A1	Cytochrome P450 family 1, subfamily A, member 1
DBP	Dibutyl phthalate
DEHP	Di(2-ethylhexyl) phthalate
ECM	Extracellular matrix
EDCs	Endocrine Disrupting Chemicals
EGFR	Epidermal growth factor receptor
EMT	Epithelial–mesenchymal transition
ER	Estrogen Receptor
ESCRT	Endosomal Sorting Complexes Required for Transport
EVs	Extracellular Vesicles
GABBR	Gamma-aminobutyric acid type B receptor subunit 1
GPER	G protein-coupled membrane estrogen receptor
HBE	Human Bronchial Epithelial cells
HCC	Hepatocellular carcinoma
IARC	International Agency for Research on Cancer
IJMS	International Journal of Molecular Sciences
ILV	Intraluminal vesicles
ISEV	International Society for Extracellular Vesicles
JNK	c-Jun N-terminal Kinase
lncRNAs	Long non-coding RNAs
miRNAs	microRNAs
MMP	Matrix Metalloproteinase
mRNAs	Messenger RNAs
MVB	Multivesicular body
NADPH oxidase	Nicotinamide adenine dinucleotide phosphate oxidase
ncRNAs	Non-coding RNAs
NF-κB	Nuclear factor kappa B
NNK	4-(methylnitrosamino)-1-(3-pyridyl)-1-butanone
OSCC	Oral Squamous Cell Carcinoma
PAHs	Polycyclic aromatic hydrocarbons
PD-L1	Programmed Death-Ligand 1
PI3K	Phosphoinositide 3-kinase
PIWI	P-element Induced Wimpy testis
PM2.5	Particulate matter 2.5
PR	Progesterone Receptor
PTEN	Phosphatase and tensin homolog
ROS	Reactive oxygen species
STAT3	Signal Transducer and Activator of Transcription 3
TME	Tumor Microenvironment
TWF1	Twinfilin-1
VEGF	Vascular endothelial growth factor
ZEB1	Zinc finger E-box-binding homeobox 1
